# Aortic hemodynamics in postmenopausal women following cessation of hormone therapy

**DOI:** 10.14814/phy2.13535

**Published:** 2017-12-06

**Authors:** Ronée E. Harvey, Maja C. Johnson, Sushant M. Ranadive, Michael J. Joyner, Brian D. Lahr, Virginia M. Miller, Jill N. Barnes

**Affiliations:** ^1^ Mayo Clinic College of Medicine and Science Mayo Clinic Rochester Minnesota; ^2^ Department of Anesthesiology Mayo Clinic Rochester Minnesota; ^3^ Department of Health Science Research Mayo Clinic Rochester Minnesota; ^4^ Department of Physiology and Biomedical Engineering Mayo Clinic Rochester Minnesota; ^5^ Department of Surgery Mayo Clinic Rochester Minnesota; ^6^ Department of Kinesiology University of Wisconsin‐Madison Madison Wisconsin

**Keywords:** Augmentation index, blood pressure, estrogen

## Abstract

Central (aortic) blood pressure and aortic pulse wave characteristics are measures of cardiovascular health, predictive of cardiovascular mortality. Previous studies have compared aortic hemodynamics in women who do and do not take menopausal hormone therapy, but characteristics of these parameters following cessation of treatment have not been defined. Therefore, the purpose of this study was to define aortic pulse wave characteristics in postmenopausal women with and without a history of menopausal hormone therapy use. Pulse wave analysis was conducted on 67 women who had participated in the randomized, double‐blind, placebo‐controlled Kronos Early Estrogen Prevention Study (KEEPS), 3 years subsequent to the four‐year treatment period. Treatment was oral conjugated equine estrogen (0.45 mg/day; *n* = 18); transdermal 17*β*‐estradiol (50 *μ*g/day; *n* = 23) each with oral micronized progesterone (Prometrium 200 mg); and placebo pills and patch (*n* = 26). At post‐treatment, median age (60 years) and body mass index (27 kg/m^2^) did not differ across prior treatment assignment. Aortic blood pressures (median systolic 115 mm Hg and diastolic 76 mm Hg) and augmentation index (median 33%) did not differ among women across prior treatment assignment. These results suggest that these doses and formulations of menopausal hormone therapy had no long‐term effects on central vascular function 3 years after cessation of treatment.

## Introduction

Cardiovascular (CV) disease is the leading cause of mortality in women (Mozaffarian et al. 2015). Although CV disease is relatively uncommon in a woman's younger years, the risk increases with age following the menopausal transition (Lima et al. 2012; Mozaffarian et al. 2015). The use of menopausal hormone therapy (HT) for the prevention of CV disease is controversial; (Langer et al. 2012; Ghazal and Pal 2013) and clinical guidelines recommend that use of HT be restricted to the lowest dose for the shortest duration needed to manage menopausal symptoms (Ghazal and Pal 2013). Additionally, treatment should begin close to the onset of menopause.

Arterial blood pressure varies from central to peripheral vessels due to differences in vascular stiffness along the arterial tree (Laurent et al. 2006; Nelson et al. 2010; McEniery et al. 2014). Due to these variations, the shape of the arterial pressure waveform also changes. The arterial pressure waveform is composed of an incident forward wave, as well as a reflected wave that is created when blood reverses flow after striking a vessel branch point (Laurent et al. 2006). In the aorta, this reflected wave can result in augmentation of systolic blood pressure, longer left ventricular ejection duration, and increased workload on the heart. Central aortic hemodynamics and pulse wave characteristics are associated with CV risk factors, such as obesity and smoking, and are important predictors of CV disease risk. At times, these assessments may be superior to peripheral brachial blood pressure measurements recorded by traditional methods, as central pressures may increase prior to peripheral pressures in early hypertension development (Laurent et al. 2006; Nelson et al. 2010; McEniery et al. 2014). Additionally, clinical treatment of CV disease is associated with a concomitant improvement in aortic hemodynamic values (Laurent et al. 2006). Noninvasive central pulse wave analysis can be used to calculate aortic blood pressures and pulse wave characteristics.

There are conflicting results regarding the effects of HT on aortic hemodynamics (Hayward et al. 1997, 2000; Tanaka et al. 1998; Tentolouris et al. 2005; O'Neill et al. 2013). It is difficult to reconcile these studies due to their varying methodologies (e.g., duration of HT, differing doses, and formulations of HT, etc.). In this context, it is unknown if aortic hemodynamics differ between women who discontinued use of menopausal HT and those who never used menopausal HT. Therefore, the purpose of this study was to evaluate aortic pulse wave characteristics in women 3 years following the use of menopausal HT compared to women who did not use HT. Because the activational effects of sex hormones (i.e., short‐term, reversible effects on vascular function) occur only in the presence of menopausal HT, we hypothesized that aortic pulse wave characteristics 3 years after cessation of the treatments would be similar between women who did and did not initiate menopausal HT.

## Materials and Methods

### Ethical approval

This study was approved by the Institutional Review Board of Mayo Clinic. All women gave written informed consent.

### Participants

Individuals were participants in the Kronos Early Estrogen Prevention Study (KEEPS) (Wharton et al. 2013). KEEPS was a randomized, double‐blind, placebo‐controlled clinical trial to test if HT initiated within 3 years of menopause slowed the progression of CV disease defined by changes in carotid intima‐media thickness. KEEPS participants were randomized to receive one of the following three treatments over a four‐year period (1) oral conjugated equine estrogen (*n* = 39; oCEE; Premarin 0.45 mg/day); (2) transdermal 17*β*‐estradiol patch (*n* = 36; tE2; Climara 50 *μ*g/day); or (3) placebo pills and patch (*n* = 43). Women in the active treatment groups also received oral micronized progesterone (Prometrium 200 mg) for 12 days each month.

For this current study, a subset of these women (*n* = 74) was evaluated 3 years following the conclusion of the KEEPS. Exclusion criteria for this study were a body mass index >35 kg/m^2^, history of CV events (i.e., stroke, myocardial infarction), diagnosis of diabetes, uncontrolled hypertension (systolic blood pressure >150 mmHg and/or diastolic blood pressure >95 mmHg on both the screen and study day visits), diagnosis of cancer, and use of medications with anti‐platelet activity and those that may influence cognitive function.

### Experimental protocol

Individuals refrained from alcohol, caffeine, and exercise for 24 h prior to the study. After fasting for four hours, participants were admitted to the Clinical Research and Trials Unit at the Mayo Clinic. Participants rested quietly in the supine position during instrumentation and throughout the study. Baseline peripheral blood pressure was established using a brachial cuff. The average of three readings for peripheral blood pressure, each separated by two minutes, was recorded. Heart rate was recorded continuously using a 3‐lead ECG (Cardiocap/5; Datex‐Ohmeda, Louisville, CO).

### Pulse wave analysis

Noninvasive assessment of arterial wave reflection characteristics was completed using the SphygmoCor system (AtCor Medical, Sydney, Australia) (Casey et al. 2011). High‐fidelity radial artery pressure waveforms were recorded by applanation tonometry of the radial pulse in the right wrist using a pencil‐type micromanometer (Millar Instruments). The radial blood pressure and waveform were calibrated from the systolic and diastolic brachial artery blood pressures. A generalized transfer function, which has been validated both intra‐arterially (Chen et al. 1997) and noninvasively (Gallagher et al. 2004), was used to generate the aortic pressure waveform.

Pulse wave analysis of the aortic pressure waveform provided the following variables of interest: aortic blood pressures; augmented pressure (AP; the difference between the first and second systolic shoulders of the aortic systolic blood pressure; that is, the amplitude of the reflected wave); aortic augmentation index (AIx); AIx adjusted for a heart rate of 75 beats/min; and wasted left ventricular pressure energy (Ew), which is the component of extra‐myocardial oxygen requirement attributable to early systolic wave reflection (Casey et al. 2011). Ew can be estimated as [(*π*/4)*(AP* Δt_r_)*1.333], where 1.333 is the conversion factor for mmHg/s to dyne•cm2•s, and Δt_r_ is the systolic duration of the reflected wave in msec. Only high‐quality recordings, an in‐device quality index of >80%, were accepted for analysis. The average of two measurements within the acceptable quality index was recorded for each individual.

### Statistical analysis

Hayward et al. (1997) reported that the absolute difference in AIx was 6.6% lower in women with current HT use compared to women who did not use HT (20.4 ± 8.6 vs. 27.0 ± 10.2%, *P* < 0.01). Due to our study design, we anticipated that the difference in AIx between women in this study would be slightly lower, as HT had been discontinued. In order to observe a difference in AIx of 4.6% between women who did not take HT and women in the HT groups (with no differences between the oCEE and tE2 groups), 153 individuals would need to be included in the analysis to obtain a power of *β *= 0.80.

To provide a description of study participants, data pertaining to demographic, clinical, and hemodynamic characteristics were presented with descriptive statistics. Quartiles (median, along with 25th and 75th percentiles) were used to summarize continuous values, while number and percentages were used to describe occurrences of categories. Differences between prior treatment groups were determined by the Kruskal–Wallis test for continuous variables and Pearson's Chi‐squared test for categorical variables. For each test, a p‐value ≤0.05 was considered significant. All analyses were conducted using SAS software, version 9.4 (SAS Institute Inc, Cary, NC).

## Results

Of the 110 women who participated in KEEPS at Mayo Clinic (Miller et al. 2009; Harman et al. 2014), 74 women agreed to participate in this study conducted 3 years after cessation of HT. Three women were excluded from the hemodynamics testing due to body mass index, and four for the use of medications that could affect testing. Clinical and demographic characteristics of the 67 participants who met inclusion criteria did not differ across prior treatment assignments (Table 1). Aortic blood pressures, augmentation index, augmented pressure, and related variables did not differ among women based on prior treatment assignment (Table 2). Removal of individuals using antihypertensive medications or current HT did not affect these results (data not shown).

**Table 1:** Demographic and clinical characteristics of participants 3 years after cessation of placebo or postmenopausal hormone therapy

Variable	Original treatment group			*P*‐value
	PL (*n* = 26)	oCEE (*n* = 18)	tE2 (*n* = 23)	
Age, yr	60 (59, 62)	61 (59, 62)	60 (59, 61)	0.731
Body mass index, kg/m^2^	27 (25, 31)	27 (24, 29)	27 (22, 33)	0.893
Heart rate, bpm	65 (54, 70)	61 (57, 63)	64 (53, 67)	0.848
Peripheral SBP, mm Hg	123 (115, 131)	123 (117, 130)	124 (119, 128)	0.916
Peripheral DBP, mm Hg	74 (67, 83)	75 (71, 79)	75 (70, 79)	0.914
Mean arterial pressure, mm Hg	88 (83, 100)	91 (86, 96)	91 (86, 95)	0.959
Low‐density lipoprotein cholesterol, mg/dL	123 (113, 144)	120 (106, 132)	117 (91, 140)	0.765
High‐density lipoprotein cholesterol, mg/dL	60 (54, 67)	64 (58, 75)	68 (56, 75)	0.473
Triglycerides, mg/dL	94 (73, 115)	104 (83, 119)	83 (69, 109)	0.395
Fasting glucose, mg/dL	94 (88, 98)	97 (92, 100)	93 (89, 100)	0.458
Insulin, *μ*IU/mL	5 (4, 7)	6 (4, 7)	4 (3, 6)	0.095
Number taking antihypertensive medications, *n* (%)	5 (19%)	3 (17%)	1 (4%)	0.280
Number taking aspirin, *n* (%)	5 (19%)	4 (22%)	4 (17%)	0.927
Number taking hormone therapy at present study, *n* (%)	7 (27%)	5 (28%)	7 (30%)	0.962
Oral, *n*	0	2	1	
Transdermal, *n*	1	1	3	
Vaginal, *n*	6	1	1	
Oral+Transdermal, *n*	0	1	1	
Buccal, *n*	0	0	1	

Data are presented as median (25th, 75th percentile), unless otherwise noted. bpm, beats per minute; DBP, diastolic blood pressure; mm Hg, millimeters of mercury; oCEE, oral conjugated equine estrogen; SBP, systolic blood pressure; tE2, transdermal 17*β*‐estradiol.

**Table 2:** Aortic hemodynamics and pulse wave characteristics of women 3 years after cessation of placebo or postmenopausal hormone therapy

Variable	Original treatment group			*P*‐value
	PL (*n* = 26)	oCEE (*n* = 18)	tE2 (*n* = 23)	
Aortic SBP, mm Hg	115 (104, 126)	114 (104, 121)	116 (111, 122)	0.756
Aortic DBP, mm Hg	76 (69, 84)	75 (70, 78)	77 (70, 80)	0.927
Pulse pressure amplification, %	119 (113, 128)	119 (114, 124)	118 (113, 122)	0.593
Augmented pressure, mm Hg	13 (9, 16)	14 (11, 17)	13 (11, 17)	0.851
AIx, %	34 (29, 40)	33 (28, 39)	33 (30, 39)	0.983
AIx at 75 bpm, %	30 (23, 33)	28 (25, 33)	27 (23, 31)	0.895
Ew, dyne•cm^2^•s	2706 (1896, 3432)	2835 (2263, 3749)	3083 (2217, 4118)	0.802
Δt_r_, msec	141 (135, 147)	139 (132, 143)	142 (130, 153)	0.514

Data are presented as median (25th, 75th percentile). AIx, augmentation index; AIx at 75 bpm, augmentation index adjusted for a heart rate of 75 beats per minute; DBP, diastolic blood pressure; mm Hg, millimeters of mercury; Ew, wasted left ventricular pressure energy; oCEE, oral conjugated equine estrogen; SBP, systolic blood pressure; tE2, transdermal 17*β*‐estradiol; Δt_r_, systolic duration of the reflected wave.

## Discussion

To the best of our knowledge this is the first study to report aortic hemodynamic and pulse wave measurements in postmenopausal women following cessation of several years of HT. These data are important because the low doses of HT used in the KEEPS represent those more typically used in clinical practice today. Additionally, there was a direct comparison between use of an oral HT formulation that contains multiple metabolites of E2 (i.e., conjugated equine estrogen) and a transdermal product containing the naturally occurring hormone (i.e., E2). The absence of a difference in hemodynamic variables following discontinuation of the HT formulations provides evidence upon which to make recommendations regarding the consequences of cessation of hormone use. These data also provide the background for additional follow‐up studies regarding the rate of change in hemodynamic parameters after longer periods (i.e., 7–10 years) of hormone cessation.

Following the publication of results from the Women's Health Initiative in 2002, HT was not recommended for the prevention of CV disease (Rossouw et al. 2002). However, the major criticism of the study was that participants included women who were over a decade beyond the onset of menopause, many of whom had already developed atherosclerosis (Langer et al. 2012; Clarkson et al. 2013; Ghazal and Pal 2013; Kreatsoulas and Anand 2013). Additionally, the generalization of results to all women regardless of age and HT formulation was not ideal. Since then, additional analyses and studies suggest that HT may not involve more than a minimal increase in absolute CV disease risk if used early after menopause, in women with low CV risk, and at a low dose for a short duration (Ghazal and Pal 2013).

Regarding CV disease risk and mortality after HT discontinuation, Mikkola et al. (2015) reported that the number of deaths attributed to stroke and coronary heart disease was greater than expected (in comparison to the standardized mortality ratio) in women during the one‐year period immediately following HT discontinuation. However, they noted that following the initial one‐year HT discontinuation period, stroke and cardiac mortality were lower than expected in women who used HT for any period of time. Those investigators suggested that acute withdrawal of estrogen may promote CV events through increased vasoconstriction (Mikkola et al. 2015). In the present study, longitudinal data were not available to determine if aortic hemodynamic factors initially worsen 1 year after HT discontinuation and then subsequently improve. Follow‐up data on adverse CV events in the entire KEEPS cohort have not been investigated, but it could be hypothesized that such events would be low, given the age and health of the women initially enrolled in KEEPS.

Results from previous studies on HT and aortic hemodynamic parameters have been conflicting with conclusions tending toward no influence of HT on these variables (Hayward et al. 1997, 2000; Tanaka et al. 1998; Tentolouris et al. 2005; O'Neill et al. 2013). A single dose of oral 17*β*‐estradiol did not alter aortic pulse wave characteristics in postmenopausal women (Hayward et al. 2000). However, two cross‐sectional studies reported that augmentation index was lower in women taking HT versus nonusers while aortic blood pressures were similar (Hayward et al. 1997; O'Neill et al. 2013). Importantly, length of HT use and type of HT were not controlled in these studies. In contrast, no differences were observed in augmentation index between women who were taking various formulations of HT for an average of 7 years and nonusers in a cross‐sectional study (Tanaka et al. 1998). Finally, in a prospective, randomized trial in which postmenopausal women received either oral conjugated equine estrogen and medroxyprogesterone acetate treatment or no treatment across 12 months (Tentolouris et al. 2005), neither augmentation index nor aortic blood pressures changed from baseline or in comparison to women who received no treatment. Taken together, aortic hemodynamic parameters are not significantly altered during HT use.

It is likely that age, time since menopause, duration of HT use, HT dosage, and presence of CV risk factors contributed to the results of the above studies. The majority of these variables have been controlled for or standardized in this current study. For the original KEEPS trial, study participants were 42–58 years old, 6–36 months postmenopausal, and free of CV disease (Harman et al. 2014). These women were randomized to an oral HT, transdermal HT, or placebo treatment; and the clinical trial was conducted in a double‐blind fashion. The treatment period lasted for 4 years and was followed by a three‐year washout period. By following strict enrollment criteria and studying this relatively healthy cohort of individuals for this current study, confounding factors were reduced, optimizing the likelihood of observing true, potential differences among treatment groups. This methodology also allowed for the assessment of the effects of HT administered during the preferred “window of opportunity,” which is the time period during which HT has a low risk for adverse CV effects but potential benefits for a woman's cognition and bone health (Sood et al. 2014). Results of this suggest that the effects of 4 years use of HT on aortic hemodynamic parameters are neutral 3 years after cessation of treatment.

Aortic hemodynamics and pulse wave characteristics are dependent on heart rate, left ventricular function, arterial stiffness, and other physical parameters (Nelson et al. 2010), HT would need to have long‐term structural changes on the heart and blood vessels in order for there to be differences among groups in this current study. Therefore, these results support the conclusion that HT initiates activational, that is, short‐term reversible effects on cellular signaling processes, such as the production of endothelium‐derived nitric oxide, other endothelial factors, matrix proteins, as well as adrenergic signaling (Arnold 2009). Moreover, any potential short‐term, structural changes in the aorta that could have occurred during the HT use period have likely waned with time; and differences among treatment groups may have been missed due to the timing of our measurements.

### Limitations

One of the limitations of this study is that aortic hemodynamic data were not available prior to randomization or at the exit visit (time of HT discontinuation) from the KEEPS. Thus, it was not possible to determine if HT had any significant effects on aortic hemodynamics at any point during the treatment period with these differences subsequently converging. Also, the medications (e.g., antihypertensive therapy) that the participants were taking may have influenced our results. However, less than 30% of individuals were taking antihypertensive medications or menopausal hormone therapies at the time of this study; and the proportion of medication use did not significantly differ across groups. Also, removal of these individuals from the analysis set did not alter the study findings. Finally, due to our fixed sample size, we may have been underpowered to detect small differences in aortic pulse wave characteristics among groups.

## Conclusions

This is the first study analyzing aortic hemodynamics in postmenopausal women from a randomized, placebo‐controlled trial in which women began HT according to the latest recommendations – soon following the menopausal transition, at a low dose, and for a short duration. These data suggest that 4 years of HT use, does not influence central aortic hemodynamics and pulse wave characteristics in postmenopausal women three years after cessation of the treatment.

## Conflict of Interest

None declared.
